# Discovery of Cyclopentane-Based Phospholipids as Miltefosine Analogs with Superior Potency and Enhanced Selectivity Against *Naegleria fowleri*

**DOI:** 10.3390/ph18070984

**Published:** 2025-06-30

**Authors:** Ahmed H. E. Hassan, Hương Giang Lê, Tuấn Cường Võ, Minji Kim, Joo Hwan No, Mohamed H. Aboutaleb, Jaehoon Sim, Byoung-Kuk Na, Yong Sup Lee

**Affiliations:** 1 Department of Medicinal Chemistry, Faculty of Pharmacy, Mansoura University, Mansoura 35516, Egypt; ahmed_hassan@mans.edu.eg; 2 Department of Pharmacy, College of Pharmacy, Kyung Hee University, 26 Kyungheedae-ro, Seoul 02447, Republic of Korea; jsim@khu.ac.kr; 3 Department of Parasitology and Tropical Medicine, Department of Convergence Medical Science, Gyeongsang National University College of Medicine, Jinju 52727, Republic of Korea; gianglee291994@gmail.com (H.G.L.); vtcuong@gmail.com (T.C.V.); 4 Institute of Health Science, Gyeongsang National University College of Medicine, Jinju 52727, Republic of Korea; 5 Department of Fundamental Pharmaceutical Science, Graduate School, Kyung Hee University, 26 Kyungheedae-ro, Dongdaemun-gu, Seoul 02447, Republic of Korea; mjsos911@naver.com; 6 Chemical and Structural Biology of Pathogens, Institut Pasteur Korea, Seongnam-si 13488, Republic of Korea; joohwan.no@ip-korea.org; 7 Pharmaceutical Chemistry Department, Faculty of Pharmacy, Horus University, New Damietta 34518, Egypt; maboutaleb@horus.edu.eg; 8 Department of Regulatory Science, Graduate School, Kyung Hee University, Seoul 02447, Republic of Korea; 9 Institute of Regulatory Innovation through Science, Kyung Hee University, Seoul 02447, Republic of Korea

**Keywords:** *Naegleria fowleri*, primary amoebic meningoencephalitis, miltefosine analogs, potential drug

## Abstract

**Background/Objectives:** *Naegleria fowleri* is a free-living amoeba that invades brain tissues causing fatal primary amoebic meningoencephalitis (PAM). An effective and tolerable therapeutic agent is still lacking. **Methods:** A series of conformationally restricted analogs of miltefosine with varied restriction positions, stereochemical configuration and lengths of alkyl chain was investigated to discover more effective and less toxic agents than miltefosine. **Results:** Among tested compounds, derivatives **2a**, **3b** and **3d** featuring 1,2- or 2,3-positional restriction with *trans*-configuration and tridecyl or behenyl alkyl chains were discovered as more potent and less cytotoxic agents. Compounds **2a**, **3b** and **3d** elicited 3.49-, 3.58- and 6.03-fold relative potencies to miltefosine and 7.53, 3.90 and 3.49 selectivity indices, respectively. Furthermore, compounds **2a** and **3b** showed IC_90_ values for *N. fowleri* lower than CC_50_ against glial C6 cells. Compounds **2a**, **3b** and **3d** induced morphological changes and programmed cell death of *N. fowleri* via the apoptosis-like pathway. The induced death of *N. fowleri* involved DNA fragmentation along with the loss of mitochondrial membrane potential. **Conclusions:** The current research presents compounds **2a** and **3b** as more potent, selective and effective agents than miltefosine against *N. fowleri* for further development.

## 1. Introduction

*Naegleria fowleri*, known as a brain-eating amoeba, is a free-living amoeba in freshwater habitats. It causes an opportunistic but severe infection of the brain called primary amoebic meningoencephalitis (PAM) [1,2,3,4]. Despite being rare, infections are devastating, showing over 97% mortality rate (only 4 of 164 known infected individuals survived in the United States from 1962 to 2022) [5]. This high mortality rate underscores the critical need for drug development to combat this parasitic infection.

*N. fowleri* infections typically occur upon exposure to contaminated freshwater bodies like lakes, rivers, hot springs or poorly maintained swimming pools, which could result in amoeba entering the nasal cavity. Once inside, it moves through the olfactory neuroepithelium, travels along the olfactory nerves, passes through the cribriform plate and finally invades the central nervous system (CNS) [3,6,7]. Reaching the brain, it causes PAM characterized by massive inflammation and haemorrhagic necrosis of the brain. Symptoms of PAM typically appear from 1 to 12 days post-infection. Unfortunately, the clinical symptoms of PAM are similar to those of viral or bacterial meningitis, including fever, nausea, vomiting and headache [6]. Subsequent symptoms may involve stiff neck, confusion, lack of awareness towards people and surroundings, seizures, hallucinations and coma [2]. Following the onset of symptoms, the illness advances quickly and typically results in death within a span from 1 to 18 days. Due to the rapid progression of the disease, early diagnosis and immediate treatment are critical for increasing the chances of survival. However, despite prompt medical care, the prognosis remains poor due to the aggressive nature of the infection.

*N. fowleri* infection is not confined solely to the United States. The most recent data show infection has been reported in 39 countries. However, the United States of America (USA), Pakistan, Mexico, Australia, the Czech Republic and India have been the most affected [8,9]. These countries are more susceptible to infection due to their warm year-round climates. A trend analysis of the PAM cases linked to contaminated water exposure in the United States indicates an expansion in the geographic range of exposure locations from the warmer southern states towards the cooler northern states, possibly because of climate change and global warming [10,11].

**Figure 1:**
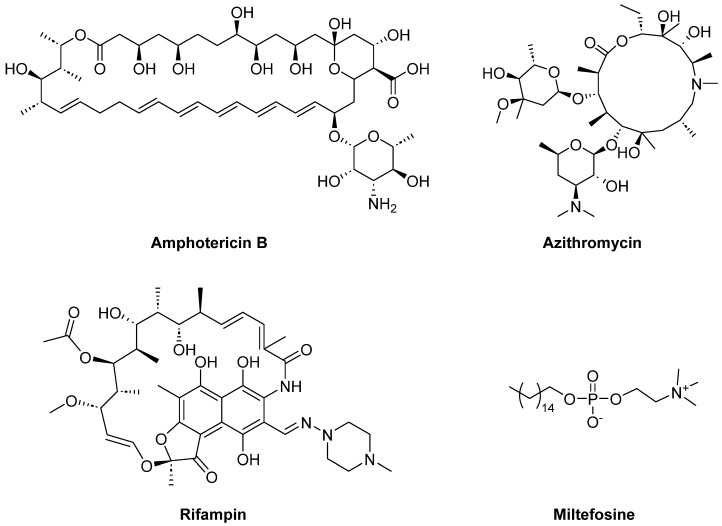
Currently used therapeutic agents for the treatment of PAM.

It is unfortunate that for such a grievous deadly infection, considerably huge gaps and obstacles in diagnosis and treatment yet exist. In addition to challenging diagnosis, the disease quickly progresses to death and, furthermore, the current recommended therapeutics are not sufficiently effective and/or suffer serious limitations [12,13]. Currently, therapeutics approaches include a drug combination of the antifungal agent amphotericin B with antibiotics azithromycin or rifampin (Figure 1) [1,14]. Recent reports have demonstrated that including the antileishmanial drug miltefosine in this therapeutic combination has successfully improved the infected patients’ chances of survival [15,16]. However, the specificity and associated toxic side effects of these drugs, such as the hepatic and nephrotoxic effects of miltefosine, remain major issues. Hence, there is an urgent need for the development of new agents against *N. fowleri* with better selectivity and lower toxic side effects.

Conformational restriction or lock is one of the well-acknowledged drug discovery and development approaches which forces flexible molecules to adopt some conformational configurations and exclude others through introducing some structural constraints [17]. This could result in favourable enhancements of activity and/or selectivity while reducing side effects and toxicities [18,19]. Meanwhile, efforts focusing on employing compounds that were either developed or that did not succeed during the development stage for treating one disease to address another disease through a process referred to as repurposing, reprofiling, repositioning or redirecting have shown numerous advantages and successful outcomes [20,21,22,23,24,25,26]. As there are the limitations and/or grievous adverse toxic effects of the currently used agents for the treatment of PAM which underscore the urgent need for the introduction of improved medications, this work was conducted, employing well-credited drug repurposing techniques towards the development of new agents for rare and neglected diseases [27,28,29,30,31,32,33] using conformationally restricted miltefosine analogues.

## 2. Results and Discussion

### 2.1. Repurposing Rational of Conformationally-Restricted Cyclopentane-Based Miltefosine Analogs

Miltefosine is a phospholipid antimicrobial drug related to lysophosphatidylchoines signalling molecules [34]. It was first used as an experimental oncology drug in the 1980s [35]. Later on, it was found efficient against *Leishmania* parasites, and its application in this regard kept evolving; in 2013, the Center for Disease Control (CDC) recommended using miltefosine as an investigational drug to treat PAM [34,36]. A promising result in surviving PAM infections has been linked to the usage of miltefosine [37,38]. This may be attributed to the ability of miltefosine to cross the blood brain barrier and concentrate in brain tissues [39]. Nevertheless, these beneficial outcomes are offset by miltefosine’s adverse effects, including hepatotoxicity, nephrotoxicity, ophthalmic toxicity, embryotoxicity, fetotoxicity and teratogenicity, which have forbid or force the discontinuation of its use [40,41,42,43,44,45,46,47]. Prompted by these facts, there is a necessity for the development of novel alternatives with superior efficacy and selectivity profiles.

**Figure 2:**
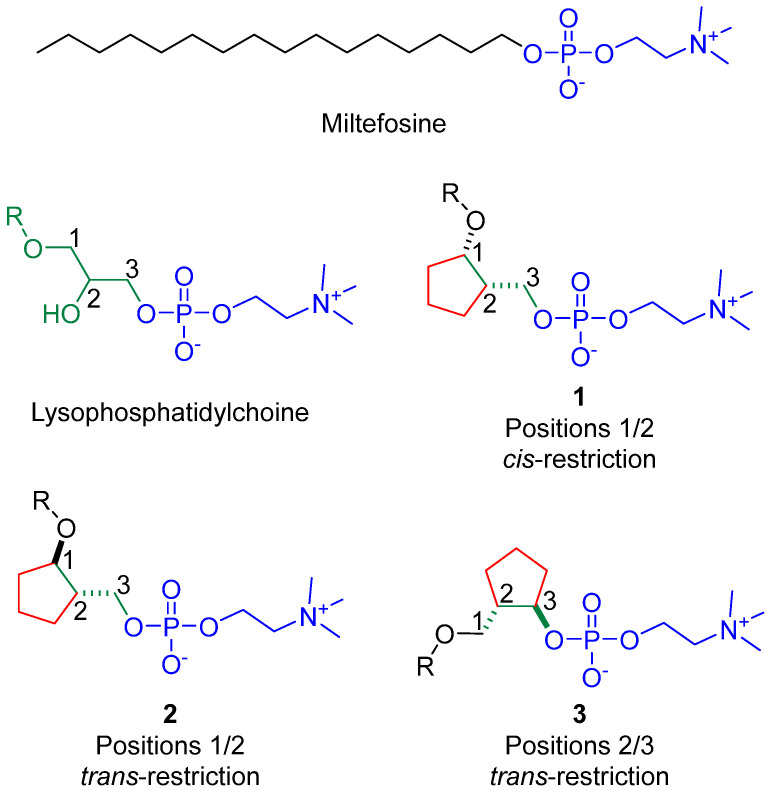
Rational underlying repurposing of conformationally restricted cyclopentane-based miltefosine analogues, where derivatives **1** are conformationally restricted at positions 1/2 of glycerol moiety of lysophosphatidylchoine in a *cis* configuration, derivatives **2** are conformationally restricted at positions 1/2 in a *trans* configuration, while derivatives **3** are conformationally restricted at positions 2/3 in a *trans* configuration.

From a structural perspective, miltefosine is a structurally flexible molecule capable of adopting numerous spatial conformations. Not all these conformers might have the same influence on favourable/unfavourable biological effects. Since certain conformers may be more associated with specific favourable/unfavourable biological effects, it could be advantageous to lock the conformational flexibility at a specific site of the molecule to try to stabilize it in a bioactive conformer-like arrangement that is more associated with the favourable biological effects. In fact, this strategy of conformational restriction has proven to be an effective drug discovery method, with documented success stories involving lipid analogues and more [48,49,50]. Therefore, the current study rational suggested that developing conformationally restricted analogues of miltefosine might help not only to enhance favourable biological effects but also could assist in enhancing selectivity and minimizing undesired toxic/adverse effects. Miltefosine was first derived from glycerophospholipids by removing the glycerol moiety located between the alkyl chain and the polar phosphocholine head (Figure 2). Considering the benefits and drawbacks of using miltefosine as an anti-*N. fowleri* agent to treat PAM, the current study rational assumed that repurposing conformationally restricted analogues of miltefosine may unveil compounds with superior selectivity and lower cytotoxicity profile. This assumption might be substantiated by the reported success of employing conformationally restricted analogues of miltefosine as antileishmanial agents, where two analogues were highly potent eliciting sub-micromolar IC_50_ values for inhibition of *Leishmania* infection compared with miltefosine [51].

To investigate these assumptions, biological evaluations of some of these constrained conformations were performed to assess their anti-*N. fowleri* effects. Targeted compounds with diverse configurations were selected to examine their effect on bioactivity. Molecules with structures **1** and **2** (Figure 2) would be suggested in this context if compounds with a removed glycerol position-2 substituent and a three-carbon bridge between glycerol positions 1 and 2 to form a cyclopentane ring confining glycerol positions 1 and 2 into a *cis* or *trans* conformation were taken into consideration. While molecules with structure **3** (Figure 2) would be suggested when compounds with a removed glycerol position-2 substituent, as well as a three-carbons bridge between glycerol positions-2 and three carbons of the glycerol moiety to form a cyclopentane ring restricting glycerol positions-2 and 3 into a trans conformation, were considered.

### 2.2. Chemistry

**Figure 3:**
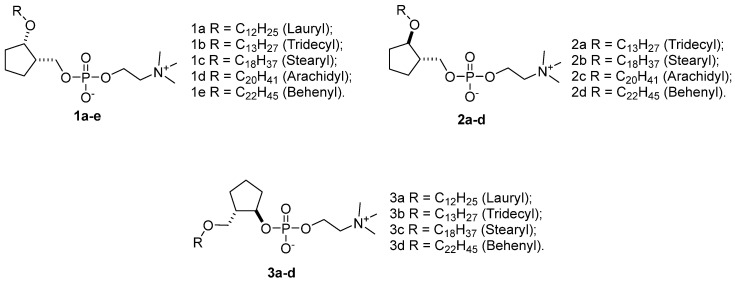
Structures of investigated compounds.

Synthesis of the targeted conformationally restricted analogues of miltefosine was previously reported by our research group [51]. The structures of the compounds investigated are illustrated in Figure 3.

### 2.3. Biological Evaluations

#### 2.3.1. In Vitro Evaluation of Anti-Amoebic Activity and Selectivity Against *N. fowleri*

A viability assay was performed to evaluate the inhibitory activity of the tested compounds by determining their IC_50_ values against *N. fowleri*, using miltefosine as a reference drug. To assess the selectivity of the tested compounds relative to miltefosine, cytotoxic values that reduced the viability of C6 glial cells by 50% were established. The results of the evaluation are presented in Table 1.

Analysis of the results showed interesting relations between the structure of the tested compounds and the elicited biological activities. While the highly flexible reference drug miltefosine was found to possess a high IC_50_ value of 146.53 µM for *N. fowleri* and a nearby CC_50_ for C6 glial cells of 158.89 µM resulting in a low selectivity index of almost 1.08, conformational restriction via the introduction of cyclopentane ring resulted, in general, in compounds eliciting superior potencies against *N. fowleri* and better selectivity indices than the highly flexible miltefosine. This might be explained by the known fact that conformational restriction confines and forces the molecules into some molecular shapes that might correspond to the bioactive conformers mediating the desired biological activity, and it excludes other molecular shapes that might correspond to conformers that possibly do not contribute desirable bioactivity or contribute to unwanted toxic effects. In addition to relative stereochemistry and the positions of conformational restriction near the phosphocholine head, the alkyl chain length was also an influential determinant of the enhancement levels in the elicited activity and selectivity. In this regard, compound **1a,** having a combination of conformational restriction at 1,2-positions with *cis*-configuration and a relatively smaller lauryl alkyl chain, did not demonstrate an enhanced potency against *N. fowleri,* showing almost 0.90-fold the potency of miltefosine (Table 1). However, the cytotoxic effect of compound **1a** against C6 glial cells was remarkably reduced, resulting in an overall increase in the selectivity index to more than 2.47. Maintaining the conformational restriction at 1,2-positions with *cis*-configuration but increasing the alkyl chain length substantiated the potency against *N. fowleri* up to C20 alkyl chain. However, this was accompanied by a general increase in the cytotoxic effects against C6 glial cells that, although apparently better than miltefosine, nullified any enhancement in selectivity index over compound **1a**. Thus, compounds **1b**, **1c** and **1d**, having tridecyl (C13), stearyl (C18), and arachidyl (C20) alkyl chains, respectively, were more potent than miltefosine against *N. fowleri* showing 3.74-, 2.01- and 3.4-fold potencies, but their selectivity indices were within a range of 1.59–2.69 (Table 1). Further increase in alkyl chain length to a behenyl (C22) alkyl chain was detrimental and resulted in compound **1e** with 0.88-fold potency of miltefosine and a low selectivity index of 0.69. Fortunately, switching the relative stereochemistry from the *cis* into the *trans*-configuration while maintaining the conformational restriction at 1,2-positions resulted in remarkable enhancements in bioactivity. In particular, compound **2a,** having a tridecyl alkyl chain, revealed 3.49-fold the potency of miltefosine with an esteemed 7.53 selectivity index. Interestingly, stearyl and arachidyl derivatives **2b** and **2c,** having *trans*-configuration, showed a 2.19- to 3.07-fold increase in potency of anti-amoebic activity relative to corresponding stearyl and arachidyl derivatives with *cis*-configuration and 6.18- and 7.46-fold potency of miltefosine. However, selectivity indices of derivatives **2b** and **2c** were not much improved relative to the corresponding *cis*-configured derivatives due to the lowering of CC_50_ values (2.94 and 2.19 versus 2.18 and 1.59 selectivity indices for *trans* and *cis*-configured stearyl and arachidyl derivatives, respectively). In the same trend, the *trans*-configured behenyl derivative **2d** was 2.98-fold more potent relative to the corresponding *cis*-configured behenyl derivative; however, it did not show much improvement in selectivity index over miltefosine. Maintaining the *trans*-configuration but translocating the conformational restriction to 2,3-positions successfully increased the potency of the long alkyl chain behenyl derivative **3d** to 6.03-fold the potency of miltefosine and 2.29-fold the potency of *trans*-configured behenyl derivative **2d** based on 1,2-conformational restriction while attenuating cytotoxicity relative to compound **2d**. Together, this resulted in an increase in the selectivity of behenyl derivative **3d** to 3.49. However, the enhancement in the potency of the *trans*-configured 2,3-conformationally restricted tridecyl derivative **3b** was minimal, while the cytotoxicity significantly increased relative to the corresponding *trans*-configured 1,2-conformationally restricted tridecyl derivative. Despite the decrease in selectivity index of compound **3b** to 3.90, it was still significantly higher than miltefosine, and all other derivatives except *trans*-configured 1,2-conformationally restricted tridecyl derivative. Meanwhile, the potencies of stearyl derivative **3c** and lauryl derivative **3a** were significantly decreased. While stearyl derivative **3c** was still more potent than miltefosine (1.92-fold the potency), it suffered a considerable impairment in selectivity index due to increased cytotoxicity. Although lauryl derivative **3a** was much less potent, possessing almost half the potency of miltefosine, it was more selective because it lacked cytotoxicity (CC_50_ > 400 µM). Overall, three cyclopentane-based conformationally restricted compounds, **2a**, **3b** and **3d,** were identified with much better potencies and selectivity indices than miltefosine. Their potencies were 3.49–6.03-fold the potency of miltefosine combined with good 3.49–7.53 selectivity indices, which represent a significant improvement over miltefosine.

#### 2.3.2. Compounds **2a**, **3b** and **3d** Induce Morphological Changes in *N. fowleri*

**Figure 4:**
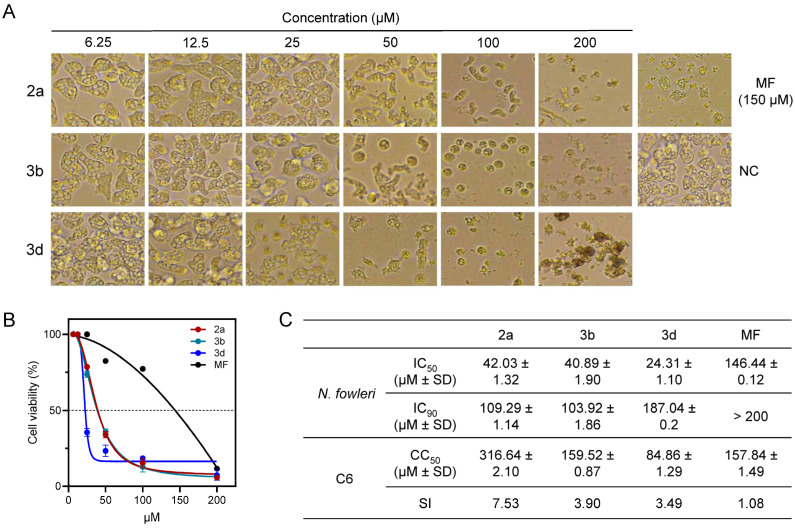
Anti-amoebic activities of compounds **2a**, **3b** and **3d** against *N. fowleri* trophozoites. (**A**) Microscopic analysis. Images represented the cell populations in three individual experiments. Miltefosine (MF: 150 μM) was employed as a positive control drug. NC, negative controls with 0.1% DMSO treatment. (**B**) Viability assay: the viabilities of amoebae and C6 glial cells are presented as a percentage relative to the untreated negative control. Results are shown as mean and standard deviation (error bar) of each assay obtained from three independent assays. (**C**) Summary. IC_50_, IC_90_, CC_50_ and SI values were calculated from three independent assays.

As IC_90_, which is the concentration that causes near complete inhibition by 90%, is an important preclinical indicator of the tissue concentration needed for clinical improvement outcome, compounds **2a**, **3b** and **3d,** which were identified as potent and selective compounds, were advanced for further evaluation (Figure 4B,C). Compounds **2a** and **3b** demonstrated close IC_90_ values of 109.29 and 103.92 µM, which is much superior to miltefosine (>200 µM). These values suggest that compounds **2a** and **3b** almost eradicated *N. fowleri* before reaching CC_50_ for C6 cells. Meanwhile, CC_50_ compound **3d** for C6 cells was lower than the IC_90_ needed to achieve the near-complete inhibition of *N. fowleri*. However, the IC_90_ value of compound **3d** was better than miltefosine (187.04 vs. >200 µM for compound **3d** and miltefosine, respectively). It is worth noting that while miltefosine has shown efficacy against *N. fowleri* in vitro, its clinical use is limited due to toxicity concerns [52]. Therefore, identifying compounds with similar efficacy but potentially improved safety profiles, such as **2a**, **3b** and **3d**, is of significant therapeutic interest. Microscopical examination showed that untreated *N. fowleri* possess the known morphological characteristics of large (from 25 to 40 μm) oval or irregular shape. On the other hand, treatment of *N. fowleri* by compounds **2a**, **3b** and **3d** induced dose-dependent morphological changes (Figure 4A). Relative to the negative control, the treated *N. fowleri* trophozoites displayed a decrease in count, accompanied by a reduction in cell size, and became a rounded shape. Such morphological changes were similar to the miltefosine-treated *N. fowleri* positive control.

#### 2.3.3. Compounds **2a**, **3b** and **3d** Induce Apoptotic-like but Not Necrotic Death of *N. fowleri*

**Figure 5:**
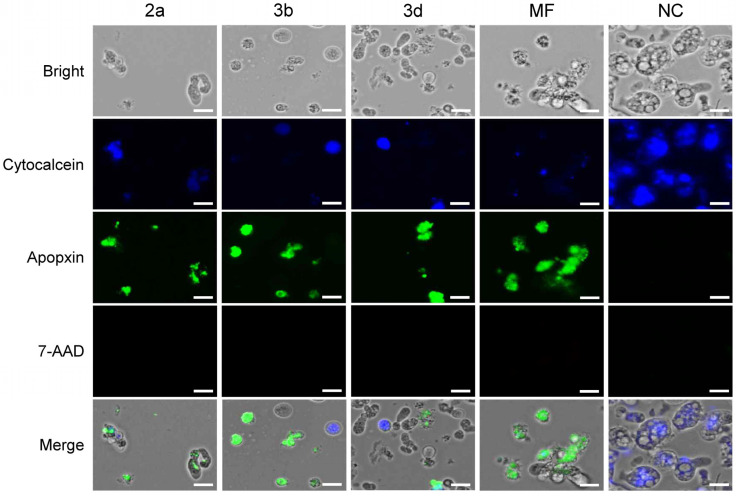
Apoptosis/necrosis assay. A fluorescence staining assay was performed using *N. fowleri* trophozoites treated with compounds **2a**, **3b**, **3d**, and miltefosine (MF) as a reference; NC, negative controls with 0.1% DMSO treatment. Blue fluorescence representing living cells stained with cytocalcein (DAPI channel, Ex/Em = 405/450 nm). Green apopxin stains apoptotic cells to show green fluorescence (GFP channel, Ex/Em = 490/525 nm). 7-Amino Actinomycin D (7-AAD) stains late apoptotic cells and necrotic cells to show red fluorescence (RFP channel, Ex/Em = 550/650 nm). Images were representatives of cell populations in three individual experiments. Size bar: 10 µm.

While apoptosis was previously believed to occur in multicellular metazoans, it was discovered that ancient forms of apoptotic-like cell death, which mimics some characteristics of apoptosis, can also trigger death in protozoa [53]. In fact, apoptosis-like death is one of the programmed cell death molecular mechanisms in protozoa that also include necrosis [54]. To further understand how compounds **2a**, **3b** and **3d** trigger the death of *N. fowleri* in comparison to miltefosine, an evaluation of apoptosis and necrosis was conducted at IC_90_ of the tested compounds for 48 h. The untreated amoebae exhibited strong blue fluorescence of the cytocalcein stain, confirming its viability (Figure 5). The observation that compounds **2a**, **3b** and **3d** induced apoptosis-like death, rather than necrosis, in *N. fowleri* is a key finding, mirroring the action of miltefosine. The diminished cytocalceine blue fluorescence, coupled with the surge in green fluorescence signifying apoptosis after treatment with the compounds (Figure 5), clearly indicates the reduction in viable cells and the initiation of programmed cell death. The absence of 7-AAD red fluorescence further reinforces the lack of detectable necrosis. This is a desirable trait for potential therapeutic agents, as necrosis, unlike apoptosis, can trigger inflammation due to the uncontrolled release of intracellular contents [39]. The similarity in the mode of cell death between these compounds and miltefosine suggests a potential convergence in their mechanisms of action, possibly targeting similar pathways or molecules within *N. fowleri* [40,41,42].

#### 2.3.4. Compounds **2a**, **3b** and **3d** Induce DNA Fragmentation in *N. fowleri*

**Figure 6:**
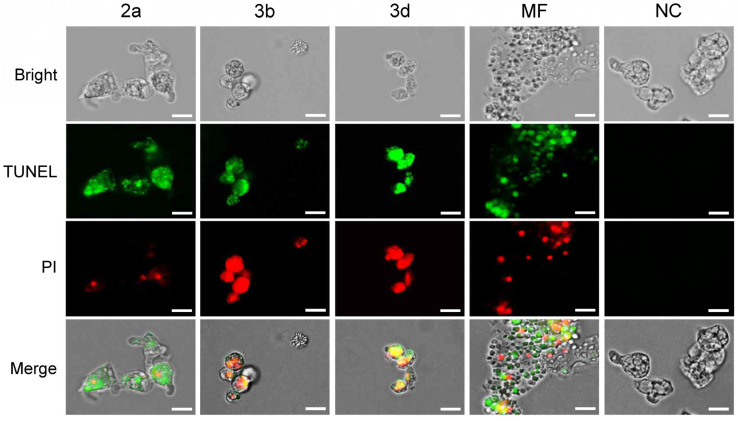
TUNEL assay. DNA fragmentation implying apoptosis was detected by TUNEL (GFP channel, (Ex/Em = 490/525 nm) in the amoebae treated with tested compounds. These amoebae were also counterstained with PI (RFP channel, Ex/Em = 550/650 nm). Miltefosine (MF) was included as a positive reference control. NC, negative controls with 0.1% DMSO treatment. Images were representatives of the cell population in three individual experiments. Size bar: 10 µm.

To further confirm the apoptotic mode of cell death induced by compounds **2a**, **3b** and **3d** in *N. fowleri*, a TUNEL assay was conducted. This assay detects DNA fragmentation, a hallmark of apoptosis, by incorporating fluorescent dUTP at DNA break sites. Fluorescence microscopy revealed no green or red fluorescence in the untreated amoebae, indicating the absence of DNA fragmentation in *N. fowleri* trophozoites (Figure 6). In contrast, treatment with miltefosine control, as well as compounds **2a**, **3b** and **3d,** resulted in intense green fluorescence, confirming DNA fragmentation and the presence of red fluorescence, indicating disrupted cell membranes (Figure 6). These results are consistent with previous studies when *N. fowleri* was treated with drugs or natural compounds [55,56,57,58]. The stronger green fluorescence observed with compounds **2a** and **3d**, compared to **3b**, suggests these compounds may induce more extensive DNA fragmentation. This difference could reflect variations in the specific molecular pathways targeted by each compound or differences in their cellular uptake or metabolism.

#### 2.3.5. Compounds **2a**, **3b** and **3d** Result in Mitochondrial Dysfunction in *N. fowleri*

**Figure 7:**
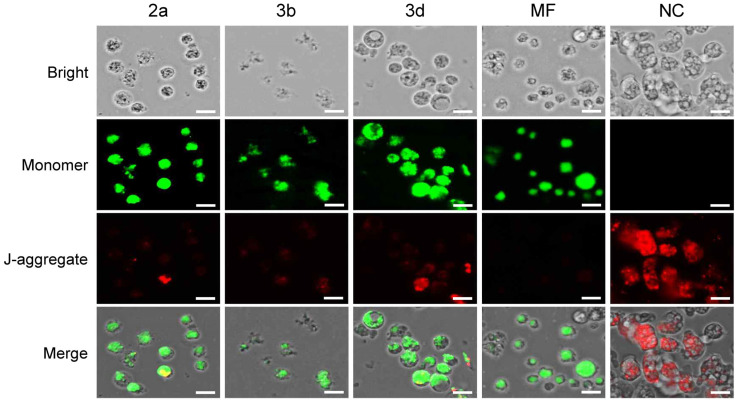
Disruption of mitochondrial functions in *N. fowleri*. Mitochondrial membrane potential changes. Aggregate form (RFP channel, Ex/Em = 550/650 nm), implying healthy mitochondria, was found in *N. fowleri* trophozoites treated with 0.1% DMSO (NC), while monomer (GFP channel, Ex/Em = 490/525 nm) indicating the collapse of mitochondrial membrane potential was increased in treated amoebae. Miltefosine (MF) was included as a positive control drug. Images were representatives of the cell population in three individual experiments. Size bar: 10 µm.

To determine whether the apoptotic death of *N. fowleri* induced by compounds **2a**, **3b** and **3d** is associated with mitochondrial dysfunction, the mitochondrial membrane potential (ΔΨm) was assessed using a JC-1 assay. The potential difference across the membrane in functioning mitochondria leads to the accumulation of cationic lipophilic fluorescent JC-1 molecules, forming red fluorescence (J-aggregate). Mitochondrial dysfunction results in the loss of mitochondrial membrane potential, leading to the transition from J-aggregates to monomers (green fluorescence). Figure 7 demonstrates the impact of compounds **2a**, **3b** and **3d** on *N. fowleri* mitochondrial membrane potential, further supporting their role in inducing apoptosis. The absence of green and strong red fluorescence in untreated amoebae indicates healthy, functional mitochondria maintaining their ΔΨm. The shift to predominantly green fluorescence upon treatment with miltefosine, as well as compounds **2a**, **3b** and **3d,** signifies a loss of ΔΨm, a critical early event in the intrinsic apoptotic pathway (Figure 7). Disruption of ΔΨm leads to the release of pro-apoptotic factors from the mitochondria, triggering the caspase cascade and, ultimately cell death [59]. This observation aligns with the previous finding of DNA fragmentation and membrane compromise, further solidifying the apoptotic mechanism of these compounds. Previous reports have also demonstrated the induction of mitochondrial damage by drugs or compounds in *N. fowleri* [60,61], suggesting a potential convergence in their mechanism of action in *N. fowleri* cell death.

## 3. Materials and Methods

### 3.1. Cells and Cultures

*N. fowleri* Carter NF69 strain (ATCC 30215; American Type Culture Collection, Manassas, VA, USA) was used. Amoeba trophozoites were cultured and maintained in Nelson’s medium containing 5% foetal bovine serum (FBS; Gibco, Grand Island, NY, USA) and 1% penicillin/streptomycin (P/S; Gibco, Grand Island, NY, USA) at 37 °C [56]. C6 rat glial cells (C6 cells; ATCC CCL-107) were cultured in Dulbecco’s Modified Eagle’s Medium (DMEM; Gibco, Grand Island, NY, USA) supplemented with 10% FBS (Gibco, Grand Island, NY, USA) and 1% P/S (Gibco, Grand Island, NY, USA) at 37 °C in a humidified incubator under 5% CO_2_ atmosphere.

### 3.2. Anti-Amoebic Activity Assays

*N. fowleri* trophozoites (5 × 10^4^ cells/well) were seeded in a 96-well microplate (Thermo Fisher Scientific, Waltham, MA, USA) in Nelson’s medium and incubated at 37 °C overnight. *N. fowleri* trophozoites were treated with 2-fold serial dilutions of tested compounds (from 0 to 200 µM) or miltefosine (150 µM) and incubated at 37 °C for 48 h. The viability of the amoebae was determined using the CellTiter-Blue^®^ Cell viability assay (Promega, Madison, WI, USA). Inhibitory concentration 50 (IC_50_) and IC_90_ were calculated by the nonlinear regression method using GraphPad Prism 9.1.0 software (GraphPad Software, San Diego, CA, USA). Amoebae treated with 0.1% DMSO were used as a control, representing 100% cell viability. Morphological changes of the amoebae were observed microscopically.

### 3.3. Cytotoxicity Assays for C6 Cells

The potential cytotoxicity of tested compounds and miltefosine in C6 cells was analysed. The cells were seeded in a 96-well microplate (Thermo Fisher Scientific; 2 × 10^4^ cells/well), respectively, and incubated overnight until 80% confluent. Serially diluted tested compounds and miltefosine were treated to the cells as described above. Morphological changes of the cells were observed by microscopic examination. Cell viability was determined using the CellTiter-Blue^®^ Cell viability assay (Promega, Alexandria, VA, USA). The cytotoxic concentrations 50 (CC_50_) of tested compounds and miltefosine were calculated using GraphPad Prism 9.1.0 software (GraphPad Software, San Diego, CA, USA). The sensitivity index (SI) was determined by the ratio between CC_50_ and IC_50_. Cells treated with 0.1% DMSO, which was confirmed not to alter the morphology of the cells by microscopic examination, were used as controls with 100% cell viability.

### 3.4. Apoptosis/Necrosis Assay

Apoptosis/necrosis in treated *N. fowleri* trophozoites was assessed using the Apoptosis/Necrosis Detection Kit (Abcam, Cambridge, UK). *N. fowleri* trophozoites (5 × 10^4^ cells/well) were seeded in a 96-well black/clear bottom plate (Thermo Fisher Scientific). Compounds **2a**, **3b** and **3d** were treated to the cells at the concentrations of IC_90_ and incubated at 37 °C for 48 h. The apoptosis/necrosis assay was performed as described previously [56]. Amoebae treated with 0.1% DMSO and miltefosine (IC_90_) were used as the negative controls (NC) and the positive controls, respectively.

### 3.5. TUNEL Assay

*N. fowleri* trophozoites (2 × 10^6^ cells/well) were seeded in a 6-well plate (Thermo Fisher Scientific), treated with compounds **2a**, **3b** or **3d** (IC_90_) and incubated at 37 °C for 48 h. The TUNEL assay was performed by using TUNEL Fluorescein Isothiocyanate (FITC) Assay Kit (Abcam, Cambridge, UK) as described previously [56]. *N. fowleri* trophozoites treated with 0.01% DMSO was used as an NC. The amoeba treated with miltefosine (IC_90_) were used as a positive control.

### 3.6. Mitochondrial Membrane Potential Assay

The electrochemical gradient changes across the mitochondrial membrane in *N. fowleri* upon treatment of compounds **2a**, **3b** and **3d** were measured using the JC-1 Mitochondrial Membrane Potential Assay Kit (Abcam, Cambridge, UK). The amoeba trophozoites (5 × 10^4^ cells/well) were seeded in a 96-well black/clear bottom plate and incubated with compounds **2a**, **3b** or **3d** (IC_90_) at 37 °C for 48 h. The JC-1 assay was conducted as described previously [56]. The amoebae treated with Nelson’s medium containing 0.1% DMSO and miltefosine (IC_90_) were used as negative and positive controls, respectively.

## 4. Conclusions

Miltefosine, the flexible molecule that was recommended by Center for Disease Control (CDC) as an investigational drug to treat PAM but suffers severe adverse effects, which might force the discontinuation of its use, was the starting point for our effort. As one conformation may mediate the desired biological function while others may produce unwanted side effects and/or toxicity, conformational restriction through the introduction of rigid cyclic structures might be helpful if it locks the flexible structure in the desired bioactive conformer and excludes those responsible for unwanted side effects and/or toxicity. In this context, we addressed rational repurposing of cyclopentane-based conformationally restrained 2-deoxy-glycerophospholipid analogues as potential and much more selective candidates than miltefosine. In vitro evaluation for their anti-amoebic activity against *N. fowleri* unveiled the high potentiality and selectivity of several members of this type of compound relative to the oral investigational drug miltefosine. Particularly, compounds **2a** and **3b** combining *trans*-configuration with positions 1,2 or positions 2,3 conformational restriction, respectively, were 3.49–3.58-fold more potent than miltefosine as inhibitors of *N. fowleri* and higher selectivity index than miltefosine (3.90–7.53 SI). Furthermore, compounds **2a** and **3b** exhibited higher CC_50_ values for C6 glial cells than their determined IC_90_ values, which eradicated amoeba infection with minimal host cells cytotoxicity, significantly improving performance compared to miltefosine. Exploration of the possible mechanism of action of compounds **2a**, **3b** and **3d** unveiled that, similar to miltefosine, they induce programmed cell death through an apoptosis-like pathway that involves DNA fragmentation along with loss of mitochondrial membrane potential. Although this study presents cyclopentane-based conformationally restricted phospholipids **2a** and **3b** as novel promising anti-*N. fowleri* agents, the current study was conducted solely in vitro, and no in vivo efficacy nor cytotoxicity investigations were addressed. Another limitation of this study is its dependence on phenotypic evaluations and lack of characterized molecular target(s). These limitations might be addressed in future studies.

## Figures and Tables

**Table 1:** In vitro evaluation results of IC_50_ values against *N. fowleri*, CC_50_ against C6 glial cells, and selectivity indices.

Compound	Alkyl Chain	IC_50_ of Inhibition of *N. fowleri*	Relative Potency to Miltefosine ^a^	CC_50_ of C6 Glial Cells	Selectivity Index ^b^
**1a**	Lauryl (C_12_H_25_)	161.98 ± 1.02	0.90	>400	>2.47
**1b**	Tridecyl (C_13_H_27_)	39.14 ± 0.60	3.74	105.37 ± 4.76	2.69
**1c**	Stearyl (C_18_H_37_)	72.73 ± 2.32	2.01	158.4 ± 0.71	2.18
**1d**	Arachidyl (C_20_H_41_)	43.64 ± 1.02	3.4	69.49 ± 0.16	1.59
**1e**	Behenyl (C_22_H_45_)	165.82 ± 068	0.88	113.65 ± 3.20	0.69
**2a**	Tridecyl (C_13_H_27_)	42.03 ± 1.32	3.49	316.64 ± 2.10	7.53
**2b**	Stearyl (C_18_H_37_)	23.72 ± 0.50	6.18	69.71 ± 0.04	2.94
**2c**	Arachidyl (C_20_H_41_)	19.63 ± 0.44	7.46	42.96 ± 8.37	2.19
**2d**	Behenyl (C_22_H_45_)	55.65 ± 3.32	2.63	71.03 ± 0.61	1.28
**3a**	Lauryl (C_12_H_25_)	279.45 ± 0.94	0.52	>400	>1.43
**3b**	Tridecyl (C_13_H_27_)	40.89 ± 1.90	3.58	159.52 ± 0.87	3.90
**3c**	Stearyl (C_18_H_37_)	76.33 ± 1.56	1.92	58.48 ± 2.08	0.77
**3d**	Behenyl (C_22_H_45_)	24.31 ± 1.10	6.03	84.86 ± 1.29	3.49
**Miltefosine**		146.53 ± 0.12	1.00	158.89 ± 1.49	1.08

^a^ Relative potency to miltefosine values were calculated by dividing IC_50_ of inhibition of *N. fowleri* of miltefosine by IC_50_ of inhibition of *N. fowleri* of each tested compound. ^b^ Selectivity index calculated by dividing CC_50_ of C6 glial cell by IC_50_ of inhibition of *N. fowleri*.

## Data Availability

All data are included in the manuscript.
